# Coping in Limbo? The Moderating Role of Coping Strategies in the Relationship between Post-Migration Stress and Well-Being during the Asylum-Seeking Process

**DOI:** 10.3390/ijerph18031004

**Published:** 2021-01-23

**Authors:** Øivind Solberg, Mathilde Sengoelge, Alexander Nissen, Fredrik Saboonchi

**Affiliations:** 1 Department of Health Sciences, Red Cross University College, 141 21 Huddinge, Sweden; mathilde.sengoelge@ki.se (M.S.); a.f.w.nissen@nkvts.no (A.N.); sabf@rkh.se (F.S.); 2 Division for Implementation and Treatment Research, Norwegian Centre for Violence and Traumatic Stress Studies, 0484 Oslo, Norway; 3 Division for Forced Migration and Refugee Health, Norwegian Centre for Violence and Traumatic Stress Studies, 0484 Oslo, Norway; 4 Division of Insurance Medicine, Department of Clinical Neuroscience, Karolinska Institutet, 171 77 Stockholm, Sweden

**Keywords:** asylum seekers, coping, stressors, mental well-being, perceived discrimination, family conflicts

## Abstract

Asylum seekers are faced with high levels of post-migratory stress due to uncertainty and uncontrollability of the application process, resulting in higher levels of mental health problems. Little is known about the coping strategies utilized by asylum seekers in this context. Structural equation modeling and the stepwise modeling approach were utilized on cross-sectional data from a cohort of asylum seekers in Sweden (N = 455) to examine whether adaptive coping in the form of problem-focused and cognitive-based coping would buffer the impact of post-migratory stressors by moderating the relationship between the stressors and well-being. Fit indices showed good to excellent fit of the final model that regressed well-being on selected post-migratory stressors and coping (CFI = 0.964, RMSEA = 0.043 (90% CI = 0.035–0.051), SRMR = 0.044). Well-being was negatively and significantly regressed on both perceived discrimination (B = −0.42, SE = 0.11, *p* < 0.001) and distressing family conflicts (B = −0.16, SE = 0.07, *p* = 0.037), and positively and significantly regressed on cognitive restructuring (B = 0.71, SE = 0.33, *p* = 0.030). There was, however, no evidence that coping strategies modified the adverse associations between the two post-migratory stressors and well-being. Interventions and policies should prioritize improving contextual factors inherent in the asylum-seeking process in order to reduce stress and enable coping.

## 1. Introduction

The asylum-seeking process has previously been referred to as a “state of limbo”, defined as a situation between two stages awaiting a decision or resolution, due to the inherent uncertainty, uncontrollability, and high levels of post-migratory stress that this temporary, transitional situation brings [1]. Previous research has also labeled the psychosocial situation asylum-seekers experience a “diathesis” or vulnerable predisposition that interacts with early post-migratory stressors, which in turn can have deleterious effects on asylum-seekers mental health [2,3]. Moreover, the prevalence of mental health disorders has been shown to be substantially higher for asylum-seekers compared to refugees who have received a formal refugee status or resident permit [3]. In fact, recent reviews have estimated that one in three asylum seekers are affected by depression, post-traumatic stress disorder (PTSD), and/or an anxiety disorder [4]. In addition, a substantial evidence base now exists that portrays the multiple risk factors faced by asylum seekers. These risk factors include pre- and peri-migration trauma, including experiencing or witnessing torture [5,6] and other forms of violence [7], multiple human rights violations and abuses during the flight to Europe, stressors upon arrival in a resettlement country [3,8], as well as a lower levels of social support during the asylum-seeking process [9]. However, considerably less focus has been directed towards the potential for adaptation in this context of trauma and adversity in the form of resilience. The concept of resilience as a psychosocial construct refers to positive adaptive characteristics to cope with and recover from adversity [10]. Consequently, adaptive coping, which has been suggested to be an individual-level underpinning factor of resilience [11,12] within this transitional, “limbo-like” situation, remains understudied.

Coping can be viewed as approaches, skills, and abilities of individuals utilized to face and manage social and environmental stressors in life in order to prevent and/or minimize stress-related difficulties or illnesses [13,14]. The role of coping in health and adaption has been widely recognized and previous literature on coping as a construct and its conceptualization is extensive [15]. Coping encompasses a large number of theoretically and empirically driven classifications [15]. The almost infinite number of possible coping responses have been divided into two overreaching categories of approach versus avoidance coping strategies [16] that distinguished the individual’s orientation towards or away from the threat or the stressors. This has become a common classification [17]. The higher-order construct of approach-based coping, further elaborated into a behavioral approach, denoting direct action and problem-solving efforts, and a cognitive approach, that includes cognitive restructuring, positive reframing, and acceptance, has thus been contrasted to coping efforts that aim to avoid and withdraw from the stressors [18]. Whereas avoidance has been consistently associated with distress and psychopathology in various populations, including those exposed to trauma [19,20], approach-based coping, such as active coping and problem solving, has since Lazarus and Folkman’s seminal paper [14], been repeatedly linked to adaptation and health [21]. Applied to the setting of an asylum-seeking process, characterized by an immense likelihood of exposure to stressors, a large set of different coping strategies might function as a moderator in the link between exposure to stressors and their adverse effects. Thus, while avoidant coping may potentially aggravate the adverse effect of stressors, adaptive approach-based coping strategies may buffer the impact of asylum-related stressors, such as the negative impact on well-being and mental health [22]. However, given the importance of contextual factors, the viability of generalizing the assumed stress-buffering functions of coping strategies in refugee population enduring asylum-seeking related stress, still needs to be investigated empirically.

Resettled refugees face difficulties in establishing and maintaining links to the host community. These range from language barriers, lack of child care needed to be able to access services or engage in activities, stigma about their religion or refugee status, and also discrimination [23,24]. Due to these challenges, some refugees may not be able to build new networks and may experience loneliness or isolation, which in turn results in depleted social networks or social support that has detrimental effects on mental health [25,26]. This might have negative consequences on their integration [27,28]. Yet, several coping strategies have been identified to date in adult refugees and asylum seekers that might assist in dealing with post-migration stressors and promote mental well-being. Social support, defined as interactions with family members, friends, peers, and professionals for information, affirmation or understanding [29], is one of the most commonly reported coping strategies utilized by refugees and asylum seekers [30]. Reaching out for support provides a sense of social connection and this is done in three ways; either via bonding with family and similar ethnic groups during the early years of resettlement [25]; or via bridging, defined as connections with other communities to assist in learning the host language and having contacts with the wider society; and thirdly, by linking with larger organizational institutions [31,32,33]. Bonding, bridging, and linking are elements of social capital, a concept that defines the existence of valued resources (capital) within, and as by-products of, social relationships and social networks [31]. Indeed, creating social network diversity has been found to be instrumental in protecting against the emergence of PTSD and to promote adjustment [34]. Engaging in social, cultural, and religious activities to promote a sense of belonging and a sense of individual and cultural identity has also been shown to be helpful. Additional factors that can provide a sense of normality, meaning, and purpose, may also include opportunities for education and training, employment and economic activities, political advocacy, and stable and secure housing [27,30,35], all factors that might be absent or restricted during the asylum-seeking process.

A growing body of knowledge is also emerging on enablers of psychological wellbeing for asylum seekers [30], but little is known on the role of coping in the asylum-seeking process in high-income countries and how such coping strategies prevent or mitigate mental health problems and/or increase well-being among asylum-seekers. The aim of this study was therefore to obtain a contextual and improved understanding of the relationship between coping and mental well-being in a population of adult, asylum-seekers currently living in housing facilities for asylum-seekers in Sweden. Our hypothesis was that given the negative impact subjective post-migratory stress has on the well-being of asylum-seekers, adaptive coping in the form of problem-focused and cognitive-based coping strategies would buffer the impact of these stressors by moderating the relationship between post-migratory stressors and well-being.

## 2. Materials and Methods

This study formed part of a larger, overarching study focusing on mental health and well-being among resettled refugees and asylum-seekers in Sweden. Parts of this methods section have therefore been previously published [3,9]. As in the aforementioned studies, the present study had a cross-sectional survey design, and data were obtained from asylum-seekers residing in three large housing facilities in Sweden. At each site, volunteers and Swedish Red Cross University College staff handed out questionnaires and consent information in the appropriate language to eligible participants. Prior to launching the study, parts of the questionnaire not already available in the appropriate languages and consent information were reviewed by a reference group of refugees with knowledge of mental health research and/or healthcare. Translation/back translation and transcultural adaptation of the questionnaire were performed and developed in collaboration with community experts in focus groups and through a pilot with 10 persons using the Think-Aloud Protocol. Interpreters/staff who were bilingual speaking persons were included in the research outreach group and were available on-site to provide assistance or information to participants in order to ensure that no language barrier existed. Eligibility was established based on asylum-seeker status, country of origin (Afghanistan, Eritrea, Iraq, Somalia, or Syria), and age (being 18 years of age or over at the time of data collection). In the present study, we use the term “asylum-seeker(s)” when referring to a person who has fled his or her home country and applied for asylum, but not yet received a residency permit, i.e., the right to international protection in a host country. The Swedish ethics committee for research approved the study.

A total of 1698 potential participants were approached from May 2016 to March 2018, and 455 respondents returned a completed questionnaire (26.8% of all 1698 eligible housing residents). The act of completing the questionnaire and handing it in was regarded as informed consent. A total of 86.9% of the respondents arrived in Sweden in 2014 or 2015 (4.5% prior to 2014, 8.6% between 2016–2018). Compared to available national statistics available from the Swedish Migration Agency (https://www.migrationsverket.se/Om-Migrationsverket/Statistik/Asyl.html) on all registered asylum-seekers in Sweden for the years 2014 and 2015, the study sample had proportionally more asylum-seekers from Afghanistan (33.8% vs. 11.9%) and Somalia (14.1% vs. 5.8%), and proportionally fewer asylum-seekers from Syria (31.9% vs. 53.3%) and Iraq (8.4% vs. 15.9%). The gender breakdown was similar in the sample compared to all asylum seekers (73.2% vs. 69.4% males, respectively), though the national statistics include minors. No comparable statistics on age were readily available, though a prior study on Syrian refugees in Sweden suggests age is positively associated with propensity for participation [8]. Thus, the sample may have proportionately fewer participants in the younger age group. See Table 1 for the characteristics of the sample.

### 2.1. Measures

#### 2.1.1. Refugee Post-Migration Stress Scale (RPMS)

Seven domains from the Refugee Post-Migration Stress Scale (RPMS) were used to measure post-migratory stressors, (1) perceived discrimination, (2) material and economic strain, (3) social strain, (4) lack of host country-specific competencies, (5) loss of home country, (6) distressing family conflicts, related to conflicts with significant others, such as a partner or other family member that cause tension and/or emotional distress, and (7) family and home country concerns, encompassing distress related to the impact of conflicts in the home country and the consequences these conflicts have on the family members [36]. The RPMS is a concise, multidimensional self-assessment instrument for the evaluation of post-migratory stress among refugees based on a theoretical conceptualization and empirical evidence of the construct of post-migratory stress. The scale consists of 21 items, all scored on a 5-point Likert scale ranging from 0 (Never) to 4 (Very often). Cronbach’s alpha was 0.83 (*perceived discrimination*); 0.76 (*material and economic strain*); 0.81 (*social strain*); 0.80 (*lack of host country-specific competencies*); 0.82 (*loss of home country*); 0.87 (*distressing family conflicts*); and 0.72 (*family and home concerns*). The 7-domain structure used has empirical support from confirmatory factor analysis of the original 24 items initially suggested [36].

#### 2.1.2. Brief COPE

Coping efforts of respondents were measured using the Brief COPE [37]. This self-report instrument is an abbreviated version of the COPE Inventory [38]. We used four scales to measure active coping, positive reframing, planning, and acceptance (two questions per scale) out of a total of 14 available scales (28 questions). We selected these subscales in order to focus on resilience-related coping, thus omitting the avoidant and social support seeking coping strategies. The eight questions were scored on a 4-point Likert scale ranging from 1 = I haven’t been doing this at all to 4 = I’ve been doing this a lot. Cronbach’s alpha was 0.70 (active coping), 0.79 (positive reframing), 0.66 (planning), and 0.66 (acceptance).

#### 2.1.3. WHO-5 Well Being Index

The respondents’ well-being was measured with the World Health Organization Five Well-Being Index (WHO-5), a short and global rating scale of current mental wellbeing (time frame the previous two weeks) [39], derived from the WHO-10 [40] based on an original 28-item scale [41]. The five-question version uses only positively phrased questions to avoid symptom-related language and is rated on a 6-point Likert scale ranging from 0 (At no time) to 5 (All the time). The total raw score, ranging from 0 to 25, is multiplied by 4 to give the final score, with 0 representing the worst imaginable well-being and 100 representing the best imaginable well-being. The instrument has demonstrated good construct validity as a unidimensional scale and has been used extensively worldwide [42]. Cronbach’s alpha was 0.94.

#### 2.1.4. Sociodemographic Variables

Data on age, country of origin, gender, and year of immigration were provided by the Swedish Migration Board. The age of respondents was categorized into 18–30 years and 31–64 years. Data on the highest level of education were self-reported.

### 2.2. Statistical Analysis

#### 2.2.1. Structural Equation Modeling

Analyses were conducted within the general framework of structural equation modeling (SEM) and the stepwise modeling approach [43]. The first step in this analytical method is to establish that measurement models of latent variables to be included in the full structural model(s) have adequate fit. This is essential to rule out that any poorly fitted full models are not due to the inadequacy of measurements. The second step consists of specifying a full structural model with hypothesized structural relationships between included observed and/or latent variables and testing the overall model fit. The present study used the following four goodness-of-fit indices to evaluate fit for both measurement and full structural models: Satorra–Bentler scaled chi-square (S-Bχ^2^); comparative fit index (CFI) with cut-off values of ≥0.90 indicating adequate fit and ≥0.95 indicating good fit [44]; root mean square error of approximation (RMSEA) with 90% confidence intervals with RMSEA < 0.06 indicating good fit, and; the standardized root mean square residual (SRMR) with SRMR < 0.08 indicating good fit [45]. Relying on several indices when evaluating model fit reduces the risk of incorrectly rejecting a well-fitted model or accepting an inadequate model based solely on information from a single index, especially given the known sensitivity of the Satorra–Bentler scaled chi-square index to model complexity and sample size [46].

Model respecifications—either to enhance model fit or to test more parsimonious models—were guided by theory and, when relevant, an inspection of modification indices. Nested models were compared using Satorra–Bentler chi-square difference likelihood ratio test (ΔS-Bχ^2^), with more parsimonious models (i.e., more constrained models with higher degrees of freedom) selected as long as the ΔS-Bχ^2^ *p*-value did not cross the 0.05 threshold indicating significantly worse model fit [47]. Non-nested models were compared using Bayesian information criterion (BIC), with smaller BIC indicating better fit [48]. Maximum likelihood estimation (MLE) with robust standard errors was used for these analyses.

#### 2.2.2. Measurement Models for SEM Methodology

Consistent with the SEM methodology, the items in the three self-report scales on post-migratory stress, coping and well-being were treated as reflective indicators of underlying latent constructs [49]. Theory and prior studies on refugee populations guided the actual modeling of latent constructs [9,50], with modification indices consulted to improve fit when necessary. The overall aim was to establish theoretically grounded and well-fitted measurement models.

Post-migratory stress was first modeled using the 7-domain structure of the RPMS identified through earlier work on Syrian refugees [36]. Malm and colleagues also suggested a second-order, 2-factor model where post-migratory stress relating to the host country are umbrellaed under the latent structure *host society stress* (comprised of the four first-order domains: *perceived discrimination*, *lack of host country-specific competencies, material and economic strain, and social strain*) and the remaining domains (i.e., *loss of home country, family and home country concerns,* and *distressing family conflicts*) are combined into the latent structure *family and home country stress*. The more parsimonious second-order 2-factor model was tested as an alternative to the first-order, 7-factor model of post-migratory stress.

Well-being was first modeled through a first-order, single-factor model with all 5 items of the WHO-5 scale included as reflective indicators of well-being. Modification indices were then inspected to evaluate potential theoretical justifiable respecifications to improve model fit.

The first coping model evaluated was a first-order, 4-factor model based on the original subscales in the Brief COPE, with both items in each two-item subscale treated as reflective indicators of that coping strategy. A more parsimonious second-order, 2-factor model based on the conceptualization of *approach coping* [16] that included the two dimensions of problem-solving and cognitive restructuring was then tested and compared to the first model [51].

#### 2.2.3. Post-Migratory Stress Direct Effect Models

As the overarching aim of the study was to examine whether the hypothesized negative effect of post-migratory stress on well-being was moderated by coping strategies, the study first set out to identify the post-migratory stressors that displayed a negative association with well-being. This was done in two steps. First, a full structural equation model was evaluated with well-being regressed on all domains of post-migratory stress, as well as age and gender. Second, a trimmed direct effect model was evaluated including only the post-migratory stressors that displayed the hypothesized negative associations with wellbeing. Since these direct effect models were non-nested, the trimmed model was compared to the full model using BIC.

#### 2.2.4. Coping Moderation Models

Moderation was examined by means of modeling latent variable interaction [52]. Latent interaction terms between selected post-migratory stressors on the basis of the results of post-migratory stress direct effect models and coping strategies were constructed and sequentially added to a baseline model which included the direct effects of significant post-migratory stressors with the addition of direct effects of coping strategies. Evaluation of moderation was then done both by direct inspection of the Wald test statistics of included interaction terms, visual inspection of interaction slopes, and, by comparing models with and without interaction using Satorra–Bentler scaled likelihood ratio (ΔS-Bχ^2^) test.

## 3. Results

### 3.1. Measurement Models and Direct Effect Models

**Figure 1:**
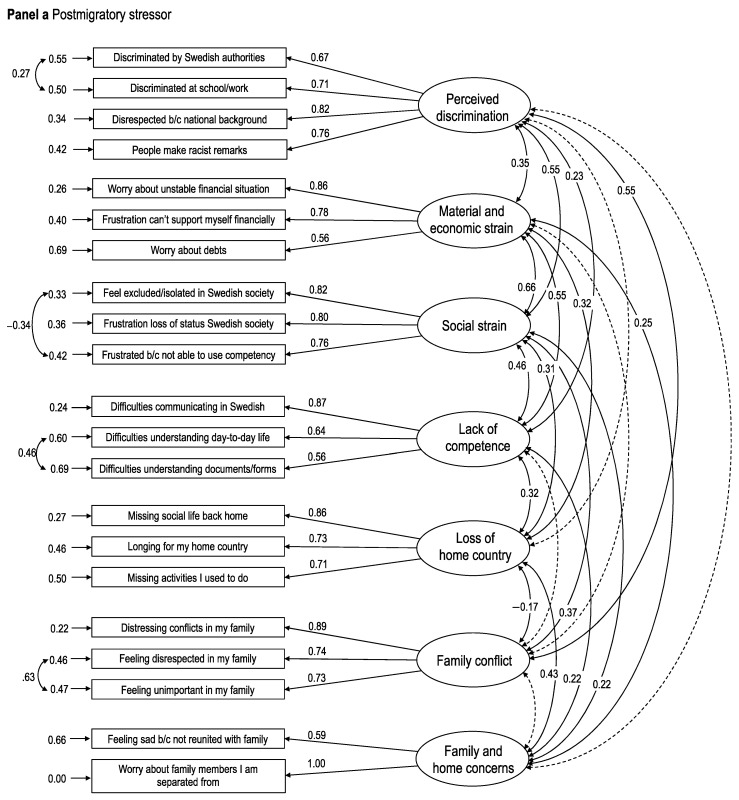

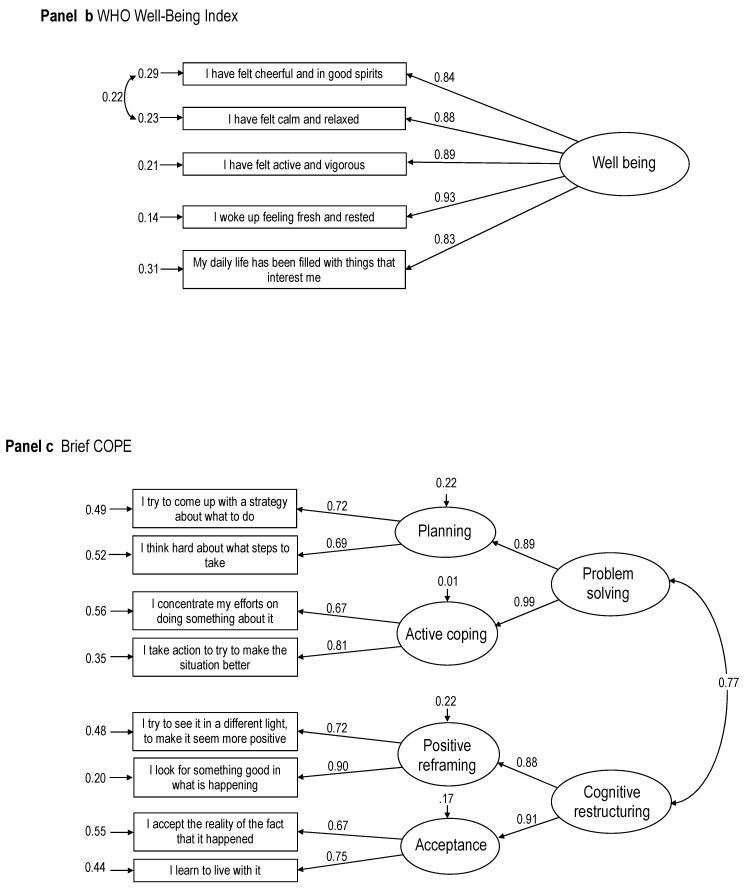
Full structural equation measurement models in the first step of the Structural Equation Model. Panel (**a**) shows the final measurement model for post-migration stressors; panel (**b**) the model for well-being; and panel (**c**) the model for coping. The displayed estimates are standardized coefficients (ß).

Table 2 summarizes fit indices for the tested measurement models. For post-migratory stress, the first-order, 7-factorial model showed a good fit across all indices except for a significant S-Bχ^2^. The more parsimonious second-order, 2-factor model was shown to not be admissible as the analyses did not converge, indicating a poorly specified model. Thus, the first-order model was selected at this step (see Figure 1a). Both the initial and respecified unidimensional measurement models for well-being showed good to excellent fit across all indices, including S-Bχ^2^. The respecified model, which included covariance between two residuals, was selected based on a significant ΔS-Bχ^2^ indicating improved model fit (ΔS-Bχ^2^ = 5.00, df = 1, *p* = 0.025) in order to minimize potential misfit in the proceeding models due to measurement modeling of the outcome variable (see Figure 1b). The initial first-order, 4-factor measurement model for coping showed adequate fit, though with an expected significant S-Bχ^2^ and an RMSEA slightly above the threshold for a well-fitted model. The more parsimonious second-order, 2-factor model had similar fit indices, and was selected as the ΔS-Bχ^2^ test comparing this model to the first-order model did not show statistical evidence of worsened fit (see Figure 1c).

The initial direct effect structural equation model regressing well-being on all post-migratory stressors showed that only perceived discrimination and distressing family conflicts were significantly and negatively associated with well-being (see Table 3, Model 1). The model also included age and gender as covariates, though neither showed significant associations with well-being. The subsequent trimmed model containing only perceived discrimination and distressing family conflicts as regressors showed good to excellent fit, and had a lower BIC compared to the initial model which contained all stressors (BIC = 14,544.9 vs. 34,060.7 for the two models, respectively) and was, subsequently, selected as input for proceeding analyses (see Table 3, Model 2).

### 3.2. Full Structural Models and Interaction

**Figure 2:**
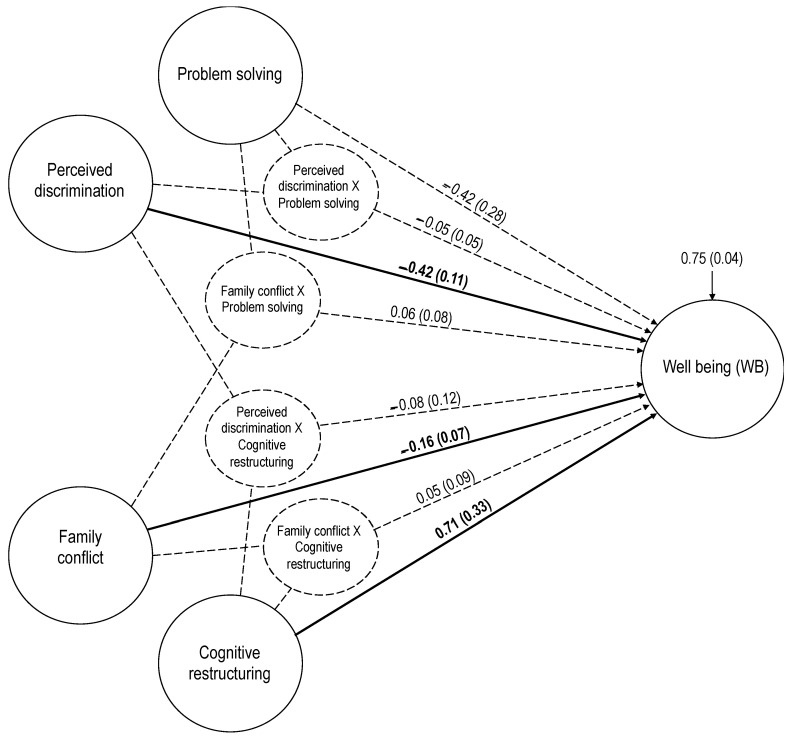
Full structural equation model of well-being regressed on post-migration stressors and coping strategies. The displayed estimates for regression weights are unstandardized (B) with robust standard error in parentheses. Significant weights are indicated by solid-line arrows. Dashed-line arrows indicate nonsignificant regression weights. The weight estimates that are significant are in bold.

The baseline full structural model regressed well-being on the post-migratory stressors *perceived discrimination* and *distressing family conflicts*, as well as the two second-order coping strategies *problem solving* and *cognitive restructuring*. Except for a significant S-Bχ^2^, the model showed good to excellent fit across the other three fit indices with RMSEA = 0.043 (90% CI = 0.035–0.051), CFI = 0.964, and SRMR = 0.044. Well-being was negatively and significantly regressed on both perceived discrimination (B = −0.42, SE = 0.11, *p* < 0.001) and distressing family conflicts (B = −0.16, SE = 0.07, *p* = 0.037), and positively and significantly regressed on cognitive restructuring (B = 0.71, SE = 0.33, *p* = 0.030), see Figure 2.

There was no evidence that coping strategies moderated the associations between post-migratory stressors and well-being. Specifically, none of the four sequentially evaluated latent interaction terms were significant in the Wald test statistics. When comparing the baseline model without interaction to the models with interaction using ΔS-Bχ^2^, there was no evidence that adding interaction improved the model. Visual inspection of the interaction slopes also corroborated a lack of moderation of the direct effects of the post-migratory stressors on well-being by coping strategies. Figure 2 summarizes the regression weights for the full structural model. Note that the regression weights for direct effects are based on the baseline model without interaction terms included, and that each interaction was evaluated separately from the baseline model.

## 4. Discussion

The aim of this study was to obtain an improved understanding of the potential role of individual coping strategies for the mental wellbeing of asylum-seekers living in housing facilities in Sweden. Furthermore, and as a result of the requisites for our analyses, the study also aimed to establish sound psychometric proprieties for measures of post-migratory stress, subjective wellbeing, and approach-based coping strategies in an asylum seeker population. Our a priori hypothesis was that adaptive approach-based coping consisting of problem-solving and cognitive restructuring strategies would buffer the impact of post-migratory stressors on psychological health. This is based on meta-analyses showing that problem coping strategies were related to improved mental health, compared to worse outcomes using maladaptive coping strategies [53]. Yet the study findings did not provide evidence of a moderating effect for neither problem-solving strategies nor cognitive restructuring on the association between these stressors and wellbeing. However, although the stress-buffering function of these coping strategies could not be supported, cognitive restructuring was positively associated with wellbeing. Two post-migratory stressors, perceived discrimination and distressing family conflicts, displayed direct adverse impacts on wellbeing scores.

The lack of stress-buffering effects of coping in the asylum-seeking situation is striking given the high levels of post-migratory stress involved and vulnerabilities stemming from potential pre-migratory trauma exposures [54]. Our results might therefore be viewed as an indicator of the profound constraints placed on asylum seekers and the depleting effect this has on their resources to cope.

It is also important to note that approximately 160,000 asylum seekers entered Sweden in 2015. As a reaction to this, the Swedish government enforced new, more restrictive asylum and reunification laws in 2016. The new regulations were quite drastic, and the aim was to reduce the number of asylum-seekers entering Sweden (https://www.migrationpolicy.org/article/sweden-turns-welcoming-and-restrictive-its-immigration-policy). These changes might have heightened the uncertainty about the future for asylum-seekers, and implied a negative change in the sociopolitical discourse and cultural attitudes towards asylum-seekers in Sweden. According to statistics from the Swedish Migration Agency, a total of 7155 individuals still awaited a decision on their asylum application as of December 2020. In 2019, 60% of the applications received a negative decision [55]. In 2019, the average processing time for applications was 9.6 months, the average stay in a housing facility was 872 days, and a total of 462 asylum seekers were in detention centers at the end of that year [56]. Although mental health outcomes are often ascribed to individual differences in coping abilities, even during extraordinary situations [57,58], our findings suggest that adaptive coping strategies [15,59] were not necessarily effective in mitigating the adverse impact of post-migratory stress in this specific asylum-seeking context.

In fact, low levels of adaptive coping have previously been shown to be related to high levels of hopelessness, helplessness, and worthlessness observed among refugees in protracted transit [60]. Problem-solving strategies, such as trying to come up with a strategy or action to improve the stressful situation [17], may therefore offer little relief for the adverse psychological impact of experiencing discrimination as an asylum seeker in a host society. Perceived discrimination may also be considered as a threat to personal and social identity [61], which has been shown to not be moderated by coping [62]. Given the transiency of the status of asylum-seekers with “foreignness” as a core identity characteristic, the set of coping responses suggested to be adopted for dealing with threats inherent in discrimination and stigma are not easily viable to asylum seekers. These include, for example, decreasing the importance of being a foreigner and a refugee attached to one’s identity [61] or self-affirmation while being constantly questioned. Repeated questioning during vetting and the asylum determination process might also intensify the feeling of being discriminated against and, in turn, have a negative impact on mental health [63]. Thus, the asylum seeker status itself may be a determinant of perceived discrimination that is fixed until the determination process has ended [64].

Distressing family conflicts may be another source of post-migratory stress that further represent a threat to personal identity [65], especially when accompanied by social changes associated with forced migration. Asylum-seekers are faced with formal impediments to achieving desired changes in social position or pre-migratory status, as well as barriers to accessing supportive social networks [66]. Thus, active coping responses may fail to buffer the impact of intra-family conflicts. Viewed through this lens, it can also be argued that confinement in small, restrictive asylum-seeker housing facilities could render “family conflicts” especially detrimental to wellbeing, or even hard/impossible to resolve, due to the uncontrollability of this stressor [67]. Cognitive restructuring, on the other hand, displayed a direct and positive association with subjective wellbeing indicating that this coping strategy may in fact be beneficial for the wellbeing of asylum-seekers in this highly tasking psychosocial situation. However, cognitive restructuring, consisting of acceptance and positive reframing coping, did not display a buffering effect for the negative impact of perceived discrimination and distressing family conflicts. These findings might further suggest that there is a lack of fit between cognitive restructuring as a coping strategy on the one hand, and post-migratory stressors that pose a threat to asylum seekers’ personal and social identities [61,65] on the other hand. This lack of fit between coping efforts and stressors has been suggested to decrease the effectiveness of coping strategies [16,68]. However, the observed positive association between cognitive restructuring and wellbeing may be attributed to this coping strategy’s fit with other sources of distress, such as trauma exposure [69]. Moreover, previous research has shown that cognitive strategies such as restructuring and reframing might generate hope for the future and positive feelings of meaning and purpose among refugees and asylum-seekers, despite the difficult aspects of the situation [30]. In summary, cognitive restructuring might be insufficient for coping with post-migratory interpersonal threats and stressors, but still have beneficial effects on the wellbeing of asylum seekers.

### Limitations

The focus of the present study was on approach-based coping strategies, such as planning, active coping, acceptance, and positive reframing, viewed as adaptive. However, other forms of coping may have been used by the asylum seekers, such as reverting to avoidance and disarmament coping in an attempt to manage the stress experienced [38]. This, in turn, may have exhausted their other coping resources [70] as they find themselves in a situation perceived as temporary and uncontrollable [71,72]. Future research using a longitudinal design should therefore explore causal relationships, including effectiveness measures and the full spectrum of coping strategies in greater detail.

The cross-sectional nature of the present data prohibits conclusive causal interpretations of the associations between post-migration stressors and wellbeing. Nevertheless, the SEM approach, built on prior studies and empirical knowledge, posits a causal structure between variables and then tests whether this structure is consistent with the data [73]. The final direct effect model in the present study showed good to excellent fit, making the posited causal structure plausible. The final moderation model was also limited in that it excluded other potential stressors related to the asylum-seeking process (e.g., fear of being sent home and lack of adequate information) as well as pre-migratory trauma history.

Lastly, the self-reported mental health measures utilized do not investigate other comorbid conditions that may produce similar symptoms or capture the complexity of all mental health dimensions. Nonetheless, the measures have been extensively used and validated to capture essential aspects of coping and well-being, while keeping questions at a minimum.

## 5. Conclusions

No evidence was found for a moderating or buffering effect of problem-solving and cognitive restructuring in the association between post-migratory stressors and wellbeing among asylum-seekers in this study. Although cognitive restructuring appears to be beneficial for the wellbeing of asylum seekers, the question remains whether general models of adaptive coping with a focus on individual-level, approach-based coping could be applied to explain differences in resilience and wellbeing outcomes among asylum-seekers. Consequently, our results highlight the inherent, structural and situational, stressors within the “limbo-like”, transitional asylum-seeking situation that may impede or deplete the effectiveness of coping resources. Future research is therefore needed in order to explore the full spectrum of coping strategies in asylum seekers in relation to mental health and well-being, especially in order to understand the interpersonal stressors, subjectively appraised as severe threats to asylum seekers’ personal and social identity, as these appear to override the potential alleviating function of approach-based coping.

## Figures and Tables

**Table 1:** Sociodemographic characteristics of the asylum seeker cohort in Sweden (N = 455).

Characteristics	*n*	%
Gender		
Women	122	26.8
Men	333	73.2
Age groups		
18–30	269	59.1
31–64	186	40.9
Educational level		
<9 years	261	57.4
9–12 years	101	22.2
>12 years	74	16.3
Missing	19	4.2
Family situation		
Living with a partner	119	26.2
Not living with a partner	261	57.4
Divorced/widow	37	8.1
Missing	38	8.4
Country of origin		
Afghanistan	154	33.8
Eritrea	45	9.9
Iraq	38	8.4
Somalia	64	14.1
Syria	145	31.9
Stateless	9	2.0

**Table 2:** Fit indices of measurement models.

Model	χ^2^	df	*p*	CFI	RMSEA (90% CI)	SRMR	ΔS-Bχ^2^	∆df	*p*
*Post-migratory stress*									
*Model A: First-order 7-factorial ^a^*	320.312	165	<0.001	0.953	0.046 (0.038–0.053)	0.055			
*WHO Well-being Index*									
Model A: Unidimensional	11.06	5	0.05	0.992	0.053 (0.000–0.096)	0.012			
*Model B: Respecified unidimensional ^b^*	4.66	4	0.32	0.999	0.020 (0.000–0.078)	0.007			
Model B vs. A							5.00	1	0.025
*Brief Cope (coping strategies)*									
Model A: First-order 4-factorial	41.82	14	<0.001	0.968	0.067 (0.044–0.091)	0.030			
*Model B: Second-order 2-factorial*	42.22	15	<0.001	0.969	0.064 (0.042–0.088)	0.030			
Model B vs. A							0.02	1	0.887

CFI Comparative Fit Index; RMSEA Root Mean Squared Error of Approximation; CI Confidence Intervals; SRMR Standardized Root Mean Square Residual; ΔS-Bχ^2^ Satorra–Bentler scaled chi Square difference; ∆df difference degrees of freedom. Selected models are in bold. ^a^ Model includes covariance between error terms of four item pairs and one error term is restrained to zero. ^b^ Model includes covariance between two error terms.

**Table 3:** Direct effect structural equation model.

	Model 1			Model 2		
	Reg. Coef.	95% CIs	*p*-Value	Reg. Coef.	95% CIs	*p*-Value
*Postmigratory stressors*						
Perceived discrimination	−0.356	(−0.581 to −0.131)	0.002	−0.498	(−0.702 to −0.294)	<0.001
Material, economic strain	−0.067	(−0.257 to 0.123)	0.489			
Social strain	−0.184	(−0.384 to 0.016)	0.071			
Lack of competence	−0.009	(−0.211 to 0.193)	0.927			
Loss of home country	0.248	(0.107 to 0.389)	0.001			
*Family conflicts*						
Family, home concerns	−0.200	(−0.347 to −0.053)	0.008	−0.220	(−0.361 to −0.079)	0.002
*Age*	0.000	(−0.261 to 0.261)	0.997			
*Gender*	−0.254	(−0.560 to 0.052)	0.103			
*BIC*	34,060.7			14,544.9		

Reg. Coef: regression coefficient; CIs: confidence intervals; BIC: Bayesian information criterion.

## Data Availability

The statistical code is available from the corresponding author. Under Swedish law and ethical approval, individual level data of this kind cannot be publicly available. Individual level data can be made available on reasonable request as long as it is in line with Swedish law and ethical approvals.
